# Supplementary material for: Exploring novel immunotherapy biomarker candidates induced by cancer deformation
Source: PLoS One. 2024 May 14;19(5):e0303433. doi: 10.1371/journal.pone.0303433 (PMC11093347; doi:10.1371/journal.pone.0303433)
Supplement: Attachment — Submitted filename: PlosOne_Response_Kim et al_Revised.pdf [file pone.0303433.s001.pdf]

Response to Reviewers

We sincerely appreciate your meticulous review of our manuscript. Your feedback is precious to us, and we have carefully incorporated your insightful suggestions into the revised version. Your thorough observations have significantly improved the quality and clarity of our research. We greatly respect the time and effort you have devoted to evaluating our work, and we eagerly anticipate receiving your esteemed feedback on the revised manuscript.

Reviewer #1:

In the present paper, authors investigated genes expressing Ig-like domains as potential biomarkers of immunotherapy in TNBC. Manuscript is potentially interesting however, an extensive english revision is needed for a better comprehension of the presented results.
Herein a few comments:

1) Results section should be more concise and should include only the authors findings; the literature data should be included in a separate section.

Response: We appreciate your valuable feedback. We have revised and improved the Results section of this manuscript accordingly. You can review the updated content in the manuscript tracking.

2) A brief paragraph on breast cancer subtype (luminal A, B, Her2 TNBC) should be included in the introduction.

Response: Thank you for your valuable comments. As per your advice, we have added content regarding the breast cancer subtype to the introduction.

3) PD-L1 pathway in TNBC as well as most relevant immunotherapy trials in breast cancers should be discussed.

Response: We are grateful for your invaluable feedback. We have incorporated the significance of PD-1/PD-L1 pathway-based treatment in triple-negative breast cancer (TNBC) into the introductory section of our results.

Reviewer #2:

Thank you for submitting the manuscript entitled "Exploring New Biomarker Candidates for Immunotherapy through Cancer Deformation-Induced Gene Changes". I was very interested in your data on MHC (HLA).

I think these findings should be published for the future research, but also have some concerns below. Hope to see you soon.

1. You present the data of only Ori and TW1 at figure 3A, 4A and 4B, but it seems you have 3

results each. Did you combine Ori1-2-3? Where did TW2 and TW3 go? Why did you focus on TW1?

Same matter on figure 3B and 4B. Why you have only TW1 and TW2? What is the differences between TW1-2-3?

Response: Thank you for your valuable feedback, and we apologize for any confusion regarding the sample names. The cells we designated as 'Ori' are MDA-MB 231 cells. Cells that underwent cancer deformation after passing through a 3μm pore size were named 'TW1,' and those that underwent further cancer deformation after TW1 were named 'TW2.' RNA sequencing experiments were conducted using Ori and TW1 cells. However, qPCR validation experiments were performed on Ori, TW-1, and TW-2. We have now made revisions in the results section to make this more transparent and less confusing.

2. If your "previous research" on page 4 and 5 were #50 on page6, please insert the reference.

Response: We sincerely appreciate your valuable feedback. Your points were accurate, and we have cited the relevant literature appropriately in the revised manuscript for your review.

3. Font of some part is different (smaller) from the rest. (page 6 and 10)

Response: Thank you for thoroughly reviewing our manuscript. We have gone through the entire formatting once again.
